# Supplementary material for: Trimetallic Nanocomposites Grafted on Modified PET Substrate Revealing Antibacterial Effect Against Escherichia coli
Source: Molecules. 2025 Dec 18;30(24):4820. doi: 10.3390/molecules30244820 (PMC12735727; doi:10.3390/molecules30244820)
Supplement: Supplementary file 1 [file molecules-30-04820-s001.zip › molecules-3991238-supplementary.pdf]

Supporting Information

Trimetallic nanocomposites grafted on modified PET substrate revealing antibacterial effect against *Escherichia coli*

Veronika Lacmanová[a], Veronika Svačinová[b], Martin Petr[c], Petr Slepička[a], Filip Průša[d], Ondřej Kvítek[a], Anna Kutová[a], Alena Řezníčková[a,*], Karolína Šišková[b,*]

[a]*Department of Solid State Engineering, University of Chemistry and Technology Prague, 166 28 Prague, Czech Republic*

[b]*Department of Experimental Physics, Faculty of Science, Palacký University Olomouc, tř. 17. Listopadu 12, 779 00 Olomouc, Czech Republic*

[c] *Regional Center of Advanced Technologies and Materials, CATRIN, Palacký University Olomouc, Šlechtitelů 27, 779 00 Olomouc, Czech Republic*

[d]*Metals and Corrosion Engineering, University of Chemistry and Technology Prague, 166 28 Prague, Czech Republic*

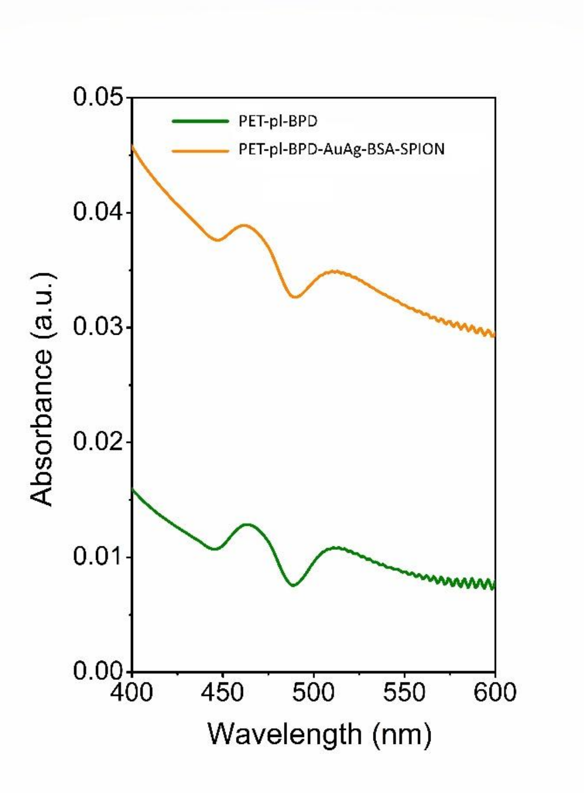

Figure S1: Comparison of UV-vis spectra of plasma-treated BPD-modified PET substrate with (orange curve) and without (green curve) grafted trimetallic nanocomposite (AuAg-BSA-SPION).

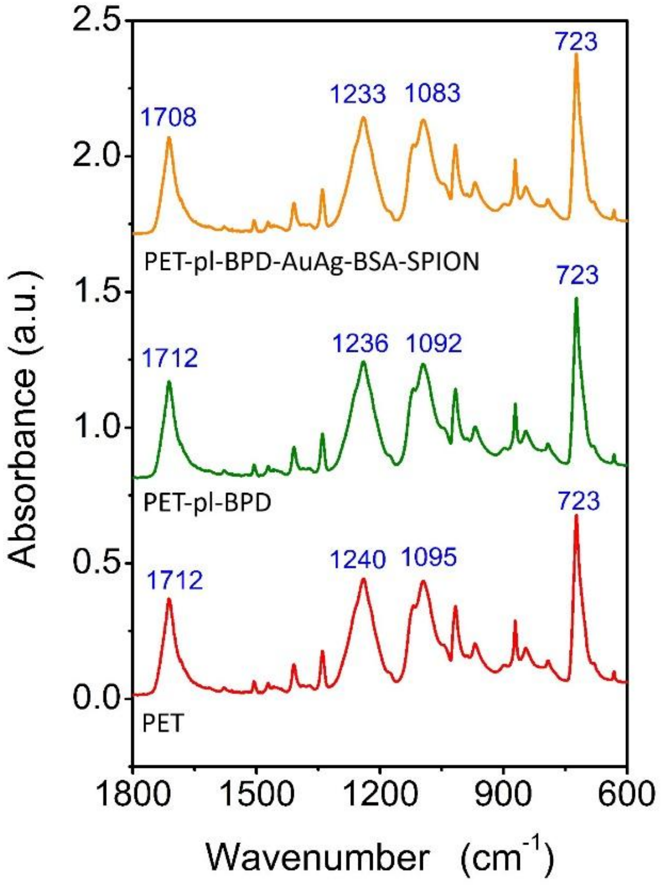

Figure S2: FT-IR spectra of PET substrate (red curve), plasma-treated BPD-modified PET (green curve), and trimetallic nanocomposite grafted on plasma-treated BPD-modified PET substrate (orange curve).

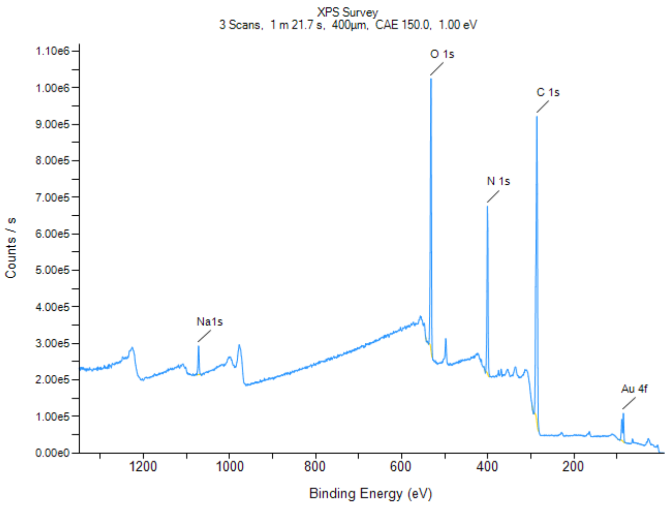

A

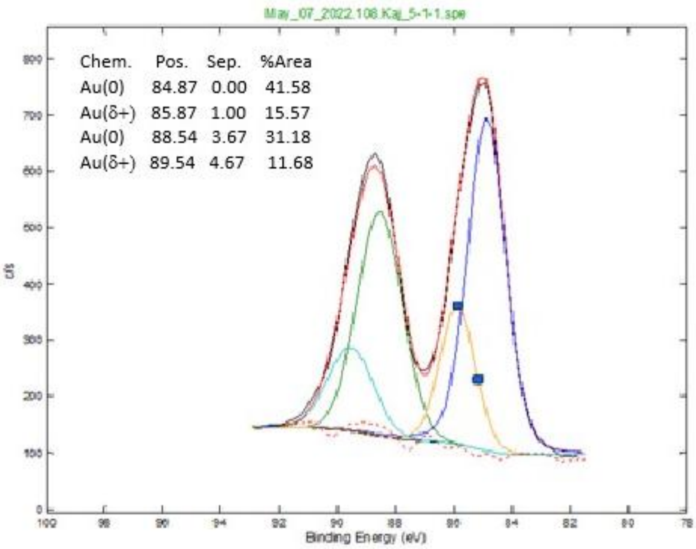

B

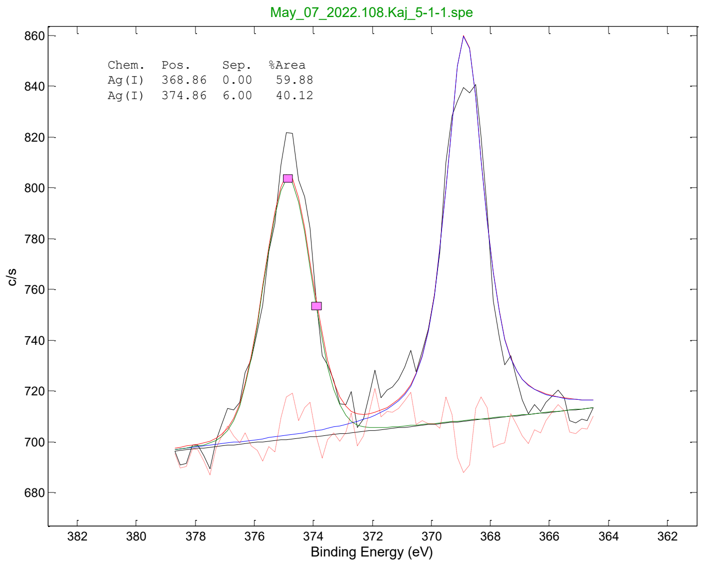

C

Figure S3: XPS survey scan from AuAg-BSA-SPION sample when drop-deposited on pl-BPD-PET substrate is shown in (A). (B) and (C) are fits of the detailed XPS signals from the trimetallic nanocomposite per se (AuAg-BSA-SPION) – namely, XPS signals of selected regions for Au 4f and Ag 3d at 0°, respectively.

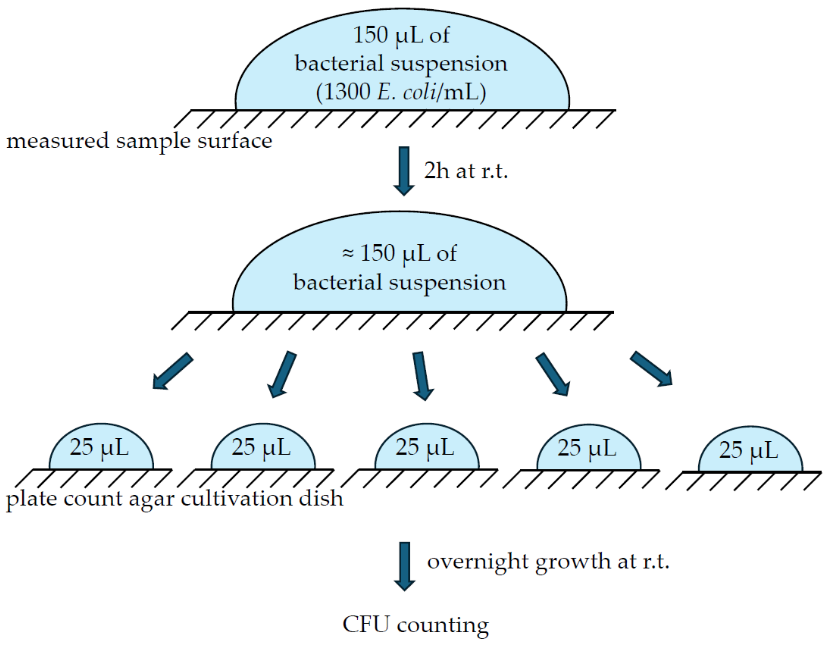

Figure S4: Schematic depiction of antibacterial testing using the drop plate method.
